# Anticipating changes in the HER2 status of breast tumours with disease progression—towards better treatment decisions in the new era of HER2-low breast cancers

**DOI:** 10.1038/s41416-023-02287-x

**Published:** 2023-04-29

**Authors:** Anthony Bergeron, Aurélie Bertaut, Françoise Beltjens, Céline Charon-Barra, Alix Amet, Clémentine Jankowski, Isabelle Desmoulins, Sylvain Ladoire, Laurent Arnould

**Affiliations:** 1 Unit of Pathology, Department of Biology and Pathology of Tumours, Georges-François Leclerc [Cancer] Centre, 1 rue du Professeur Marion, 21000 Dijon, France; 2 Unit of Methodology and Biostatistics, Georges-François Leclerc [Cancer] Center, 1 rue du Professeur Marion, 21000 Dijon, France; 3 Department of Surgery, Georges-François Leclerc [Cancer] Center, 1 rue du Professeur Marion, 21000 Dijon, France; 4 Department of Medical Oncology, Georges-François Leclerc [Cancer] Center, 1 rue du Professeur Marion, 21000 Dijon, France; 5 INSERM U1231, 7 boulevard Jeanne d’Arc, 21000 Dijon, France; 6 University of Burgundy-Franche Comté, 32 avenue de l’Observatoire, 25000 Besançon, France

**Keywords:** Breast cancer, Molecular medicine, Predictive markers, Cancer therapy

## Abstract

**Background:**

HER2 expression is often negative or low in primary breast cancers (BCs) but its changes with disease progression remain poorly known. We aimed to estimate them between primary and recurrent tumours, and identify predictive factors.

**Methods:**

We compared the HER2 status, and clinical and pathological characteristics by its evolution category (stable or changed), between all primary BCs and matched recurrences registered in our database in 2000–2020 (*n* = 512).

**Results:**

HER2-low tumours were the most prevalent at diagnosis (44.9%), followed by HER2-negative tumours (39.3%). HER2 status significantly changed in 37.3% of recurrences, mainly of HER2-negative and HER2-low tumours. HER2-negative tumours which relapsed as HER2-low significantly more frequently expressed oestrogen receptors (ER) and recurred later than stably HER2-negative tumours. Changed HER2 status in distant metastases correlated with lower proliferation rates and higher ER expression in primary tumours, and among metastases of hormone receptor-positive (HR+) tumours—with weak progesterone receptor (PR) expression in primary tumours.

**Conclusions:**

HER2 status changes with BC progression, with enrichment of HER2-low tumours in advanced stages. The ER+/PR− status, low proliferation index and time to late recurrence correlated with these changes. These findings highlight the need of retesting recurrences, especially of HR + primary tumours, to identify candidates for new anti-HER2 therapies.

## Introduction

Breast cancer (BC) is the most commonly diagnosed cancer in women worldwide [1]. It is a heterogeneous disease that encompasses distinct tumour subtypes with different biological characteristics and different prognoses. The development of transcriptomic studies in the early 2000s made it possible to propose an intrinsic molecular classification of breast cancers into five main subtypes defined by the levels of expression and the co-expression of certain genes as well as by their prognosis [2, 3]. However, this classification is not yet used as such in routine clinical practice. To date, treatment decisions are taken based on the results of conventional histopathological and immunohistochemical analyses (ER, PR, HER2 and Ki67) identifying four main “surrogate” BC subgroups: luminal A, luminal B, Human epidermal growth factor receptor 2 (HER2)-positive, and triple-negative BC (TNBC). This classification has both prognostic and predictive value in clinical practice [4–7].

Among these four subgroups, HER2-positive tumours represent ~15% of all BCs [8]. This subtype is defined by the amplification of the *ERBB2* gene, a strong oncogenic driver, which leads to the HER2 receptor overexpression [9]. HER2-positive tumours are particularly aggressive and have a poor prognosis in the absence of treatment [2, 10–12]. However, since the early 2000s, the development of new therapeutic strategies specifically targeting the HER2 activation has significantly transformed the management and improved the prognosis of patients with these cancers [13, 14]. For approximately fifteen years, the identification of patients eligible for anti-HER2 treatments in clinical practice has followed a binary rule: HER2-positive status (as defined by 3 + HER2 immunohistochemistry staining or 2 + HER2 staining with in situ hybridisation (ISH) positivity (gene amplification)) has been considered as an indication for targeted anti-HER2 therapy, while HER2-negativity (as defined by 0 and 1+ staining, or 2+ staining with ISH negativity) excluded this type of treatment. This stratification is maintained in the latest international recommendations of the College of American Pathologists (2018 ASCO/CAP HER2 testing guidelines update) [15].

This dichotomous classification into HER2-positive and HER2-negative BCs, very useful in the context of anti-HER2 therapies, has currently been shaken up since new anti-HER2 therapies (antibody-drug conjugates, ADC) were shown to be effective in patients with HER2 2+ unamplified and HER2 1+ tumours, which were to date considered as HER2-negative [16–18]. These tumours, recently referred to as « HER2-low », whether luminal or triple-negative, could account for up to 55% of all BCs [19, 20]. The recent development of ADC therapies thus offers fervent hope for the large proportion of patients whose tumours fit the definition of this novel entity.

Following the emergence of these new therapies, HER2-low tumour characterisation has been the focus of many recent studies reported in the literature. However, little is known about whether and how HER2 status in tumours from the HER2-low category evolves with the disease progression and so whether it may differ between the primary and recurrent breast tumour in the same patient [21, 22]. And although the HER2-positive status is rather stable during the course of the disease, it does not seem to be the case for HER2-low and HER2-negative breast tumours [21, 22].

In order to close this gap, in this study, we aimed to characterise the HER2 status evolution with the disease progression, by comparing it between the primary tumour and a matched recurrence (local recurrence or distant metastasis), as well as to identify possible clinical and pathological factors associated with these changes.

## Materials and methods

### Study design, patient selection and data

The study group comprised all breast cancer patients diagnosed at the Department of Pathology in the Georges-François Leclerc Cancer Centre (CGFL, Dijon, France) between 2000 and 2020 for whom the HER2 status was available for both primary tumour and a matched recurrence (local recurrence or distant metastasis; *n* = 512). Patients with a prior history of breast cancer and/or contralateral breast disease at diagnosis were excluded.

Data on patient’s age at the time of diagnosis, tumour size, multifocality, histological subtype, glandular differentiation, nuclear grade, mitotic count, lymph node status, disease stage and recurrence information (timing and site of relapse) were retrieved from medical records for all subjects. The clinical, pathological and immunohistochemical characteristics of primary tumours were compared between groups differing in the HER2 status evolution (stable versus changed HER2 status in relapse).

CGFL has been authorised to conduct scientific research by relevant French authorities (authorisation number AC-2019-3531). As per our institutional policy, informed consent was obtained at diagnosis (initial biopsy) or at surgery (for patients diagnosed elsewhere) from all patients participating in the study. The study was approved by the CGFL Ethical and Scientific Committee.

### Tumour characteristics

For patients who underwent surgery before adjuvant systemic therapy (without neoadjuvant systemic therapy), the tumour size was determined using the pT values which had been defined according to the latest classification by the American Joint Committee on Cancer Staging (AJCC, 8th Edition) and recorded in surgical pathology reports. For the others, the size was determined based on ultrasound scan reports. Multifocality was defined by the presence of at least two distinct tumours on macroscopic examination, as reported in surgical pathology reports for patients who underwent primary surgery without neoadjuvant systemic therapy, or determined based on ultrasound scan reports (for the others). For all subjects, the three parameters used in the Elston & Ellis grading system were jointly re-scored by a pair of pathologists on pre-chemotherapy samples and the TNM stage was re-assigned using the latest classification by the AJCC (8th Edition).

### Determining the hormone receptor and HER2 status

Both oestrogen receptor (ER) and progesterone receptor (PR) status were retrieved from pathology reports and were classified as positive in case of positive immunostaining in at least 10% of invasive tumour cells. Tumours with a score between 1 and 9% were considered as negative. The choice of this cut-off is supported by the fact that these tumours present biological and transcriptomic profiles very close to HR-negative tumours [23]. The hormonal receptor (HR) status was considered to be positive in case of ER and/or PR positivity, while it was classified as negative in case of negativity of both ER and PR.

The HER2 status data, which had been evaluated according to ASCO/CAP recommendations in place at the time of diagnosis, were also retrieved from pathology reports. The HER2 status was considered to be positive in case of score 3+ by immunohistochemistry (IHC) and/or 2+ with fluorescence in situ hybridisation (FISH) positivity. Tumours with negative IHC staining (0) were considered as negative, whereas those with a 1+ IHC or a 2+ IHC combined with FISH negativity, previously classified as HER2-negative, were now re-classified as HER2-low.

### Statistical analysis

Continuous variables were expressed as numbers of observations, means (with standard deviation) and medians (with min–max), and categorical variables as frequencies and percentages. Comparisons between two groups were done using the Student’s or Wilcoxon test, depending on the normality of the distributions for quantitative variable and the Chi^2^ or Fisher test for qualitative ones. Comparisons between the three HER2 status groups were performed using ANOVA, Wilcoxon, Chi^2^ or Fisher tests, as appropriate. Then, post hoc tests were conducted to identify pairwise differences for variables with *P* values below 5% in the main analyses, using the Bonferroni method to take into account the alpha risk inflation. Sankey diagrams were built to summarise the evolution of the HER2 expression from primary to recurrent BC. Cohen’s kappa coefficient (K) was used to evaluate the concordance of the HER2 status between primary and recurrent tumours. Independent predictors of the HER2 status evolution were determined using Cox regression univariate and multivariate analyses. Co-variables with *P* values below 0.200 in univariate analysis were included in the multivariate model, while variables with more than 20% of missing data were excluded. Hazard ratios were determined with 95% confidence intervals. Tests were two-sided, and the significance threshold was set at 5%. All statistical analyses were performed using the SAS software, version 9.4, with the exclusion of missing values.

## Results

### Patients and tumours

A total of 512 patients were eligible for the study. Patients were all women, with the mean age of 55.8 years (range: 25–89 years). Among those, 165 patients (32.2%) presented with Stage I disease, 179 (35.0%)—Stage II, 128 (25.0%)—Stage III, and 40 (7.8%) with Stage IV disease. Almost three-quarters of patients (367 patients; 71.7%) patients presented with distant metastases while 145 (28.3%) had local recurrences. The median time between diagnosis and relapse was 60.0 months (range: 1–240 months): 77.0 months for local recurrence (range: 6–235) and 60.0 months for distant metastasis (range: 1–240).

At diagnosis, 365 (71.3%) patients presented with HR-positive tumours (luminal-like), 81 (15.8%) with HER2-positive BCs (HR-positive or -negative) and 60 (11.7%) with TNBCs. The tumour HR status was unknown for 6 (1.2%) patients. Among cases previously defined as HER2-negative (i.e., HER2 IHC 0, 1 +, or 2+ with no HER2 amplification by FISH), 230 (44.9%) primary tumours were HER2-low and 201 (39.3%) were really HER2-negative (IHC 0). HR-positive BCs were more frequently HER2-low at diagnosis than HR-negative BCs (48.1% versus 26.7%; *P* < 0.001).

Detailed demographic, clinical, and histological data of patients and tumours at diagnosis for the three HER2 status categories are shown in Tables 1 and 2. Compared to HER2-negative (IHC 0) tumours, HER2-low tumours were significantly larger (mean size: 2.8 cm versus 2.5 cm; *P* = 0.013) and more frequently expressed both HR (ER: 89.3% versus 81.0%; *P* = 0.015; and PR: 76.7% versus 66.7%; *P* = 0.025). The lobular histological subtype was more common among HER2-negative and HER2-low tumours than among HER2-positive tumours (22.4% and 19.1% versus 2.5%; *P* = 0.009 and <0.001, respectively). In contrast, in the HER2-positive tumour group, the Elston and Ellis grade was higher than in HER2-negative and HER2-low tumours (*P* = 0.002 and 0.003), HR expression was less frequent (*P* < 0.001) and relapses occurred earlier (after 58.1 months versus 78.1 for HER2-negative and 72.2 for HER2-low tumours; *P* = 0.007 and 0.039, respectively). No differences in age at diagnosis, tumour glandular differentiation, or mitosis score were observed between the three groups.

**Table 1:** Clinical and pathological characteristics of the study population and tumours according to the HER2 status of the primary tumour at diagnosis.

		HER2 status at diagnosis			
	All (*n* = 512)	HER2-negative (*n* = 201)	HER2-low (*n* = 230)	HER2-positive (*n* = 81)	*P* values
Age at diagnosis (years)					*0.060*
Mean ± SD	55.8 ± 12.9	55.7 ± 12.8	56.8 ± 12.9	53.2 ± 13.1	
Median [min–max]	56.0 [25.0–89.0]	57.0 [26.0–89.0]	57.0 [25.0–86.0]	52.0 [31.0–81.0]	
Unifocal tumour					***0.034***
Yes	397 (77.5%)	165 (82.1%)	177 (77.0%)	55 (67.9%)	
No	115 (22.5%)	36 (17.9%)	53 (23.0%)	26 (32.1%)	
Histology					***<0.001***
IDC	412 (80.5%)	149 (74.1%)	184 (80.0%)	79 (97.5%)	
ILC	91 (17.8%)	45 (22.4%)	44 (19.1%)	2 (2.5%)	
Other	9 (1.8%)	7 (3.5%)	2 (0.9%)	0 (0.0%)	
Tumour size (cm)					***0.008***
Mean ± SD	2.7 ± 1.9	2.5 ± 1.9	2.8 ± 1.7	3.0 ± 2.1	
Median [min–max]	2.2 [0.1–10.5]	2.0 [0.1–10.5]	2.5 [0.1–10.0]	2.5 [0.2–10.0]	
Missing data	6	4	1	1	
E&E grade					***0.007***
I	83 (16.4%)	40 (20.3%)	40 (17.6%)	3 (3.7%)	
II	264 (52.3%)	97 (49.2%)	123 (54.2%)	44 (54.3%)	
III	158 (31.3%)	60 (30.5%)	64 (28.2%)	34 (42.0%)	
Missing data	7	3	3	1	
Glandular differentiation					*0.320*
I	7 (1.4%)	5 (2.5%)	2 (0.9%)	0 (0.0%)	
II	146 (28.9%)	52 (26.3%)	73 (32.2%)	21 (26.3%)	
III	352 (69.7%)	141 (71.2%)	152 (67.0%)	59 (73.8%)	
Missing data	7	3	3	1	
Nuclear grade					***<0.001***
I	18 (3.6%)	14 (7.1%)	4 (1.8%)	0 (0.0%)	
II	330 (65.3%)	126 (63.6%)	163 (71.8%)	41 (51.2%)	
III	157 (31.1%)	58 (29.3%)	60 (26.4%)	39 (48.8%)	
Missing data	7	3	3	1	
Mitosis score					*0.105*
I	237 (46.9%)	93 (47.0%)	117 (51.5%	27 (33.8%)	
II	124 (24.6%)	50 (25.3%)	50 (22.1%)	24 (30.0%)	
III	144 (28.5%)	55 (27.8%)	60 (26.4%)	29 (36.3%)	
Missing data	7	3	3	1	
Lymph node status					***0.030***
N0–N0i+	235 (45.9%)	106 (52.7%)	102 (44.3%)	27 (33.3%)	
N1–N1mic	188 (36.7%)	60 (29.9%)	89 (38.7%)	39 (48.1%)	
N2–N3	89 (17.4%)	35 (17.4%)	39 (17.0%)	15 (18.5%)	
Disease stage					***0.001***
I	165 (32.2%)	79 (39.3%)	68 (29.6%)	18 (22.2%)	
II	179 (35.0%)	62 (30.8%)	88 (38.3%)	29 (35.8%)	
II	128 (25.0%)	50 (24.9%)	59 (25.7%)	19 (23.5%)	
IV	40 (7.8%)	10 (5.0%)	15 (6.5%)	15 (18.5%)	
Oestrogen receptor expression					***<0.001***
Positive	413 (81.6%)	162 (81.0%)	201 (89.3%)	50 (61.7%)	
Negative	93 (18.4%)	38 (19.0%)	24 (10.7%)	31 (38.3%)	
Missing data	6	1	5	0	
Progesterone receptor expression					***<0.001***
Positive	322 (66.4%)	124 (66.7%)	168 (76.7%)	50 (67.5%)	
Negative	163 (33.6%)	62 (33.3%)	51 (23.3%)	30 (37.5%)	
Missing data	27	15	11	1	
Time to recurrence (months)					***0.010***
Mean ± SD	72.3 ± 50.8	78.1 ± 53.6	72.2 ± 49.1	58.1 ± 45.9	
Median [min–max]	60 [1.0–240.0]	60 [1.0–240.0]	62 [1.0–235.0]	48 [1.0–182.0]	

*E&E* Elston and Ellis, *HER2* human epidermal growth factor receptor 2, *IDC* invasive ductal carcinoma, *ILC* invasive lobular carcinoma, *SD* standard deviation.

Values in bold italic show statistically significant *P*-values.

**Table 2:** Post hoc tests for clinical and pathological characteristics which were found to significantly differ between the HER2-negative, HER2-low, and HER2-positive primary breast tumours using the Bonferroni method.

	HER2-negative vs. HER2-low tumours	HER2-negative vs. HER2-positive tumours	HER2-low vs. HER2-positive tumours
Unifocal tumour			
* P* values	*0.107*	***<0.001***	***<0.001***
Histology			
* P* values	*0.189*	***0.009***	***<0.001***
Tumour Size			
* P* values	***0.013***	*0.055*	*0.919*
E&E grade			
* P* values	*0.581*	***0.002***	***0.003***
Nuclear grade			
* P* values	***0.015***	***0.001***	***<0.001***
Lymph node status			
* P* values	***0.024***	***0.033***	*0.169*
Disease stage			
* P* values	*0.162*	***<0.001***	***0.016***
Oestrogen receptor expression			
* P* values	***0.015***	***<0.001***	***<0.001***
Progesterone receptor expression			
* P* values	***0.025***	***<0.001***	***<0.001***
Time to recurrence			
* P* values	*0.643*	***0.007***	***0.039***

*E&E* Elston and Ellis, *HER2* human epidermal growth factor receptor 2.

Values in bold italic show statistically significant *P*-values.

### Differences in the HER2 status between primary and recurrent tumours

**Fig. 1:**
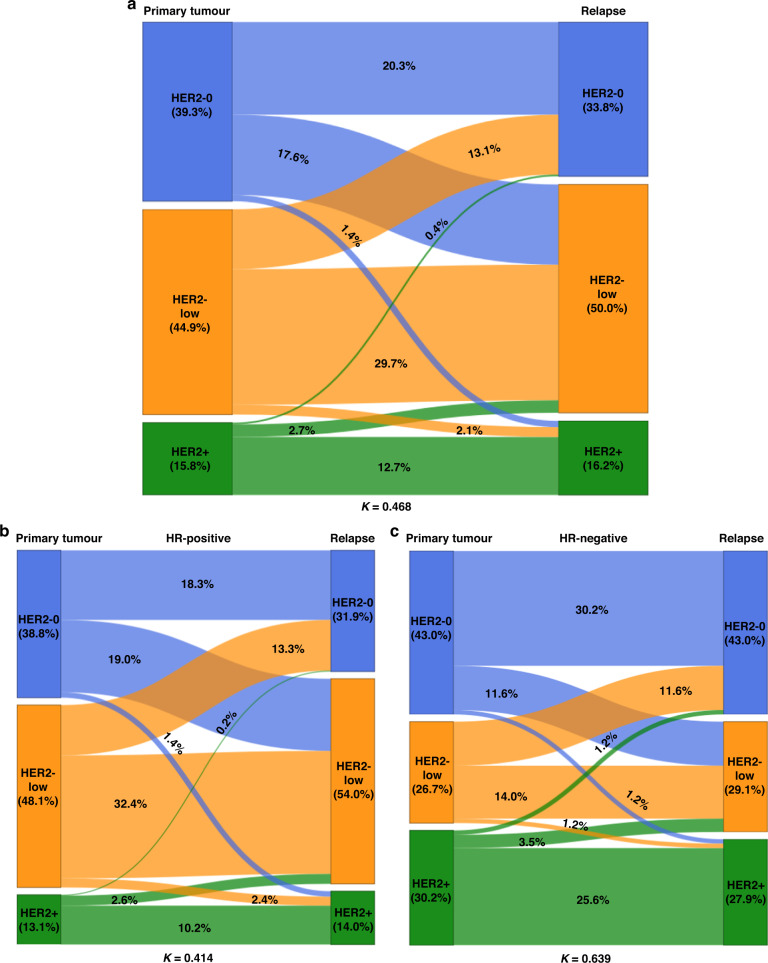
The evolution of the HER2 status between primary breast tumour and recurrence, stratified by the hormone receptor (HR) expression. Sankey diagrams illustrating the HER2 status changes for all cases (*n* = 512) (**a**), in HR-positive (HR+) (**b**) and HR-negative (HR−) (**c**) breast tumours, with specific percentages. HER2-0 HER2-negative.

**Fig. 2:**
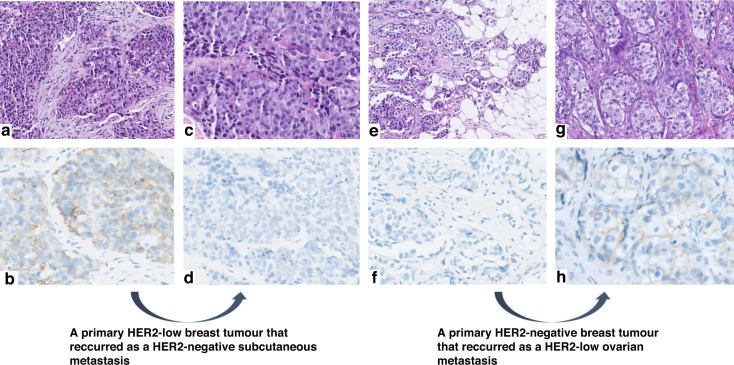
Two examples of HER2 status evolution between primary invasive breast carcinomas and their respective metastases. High-power views of two primary breast carcinomas and their metastases, with corresponding HER2 immunostaining (IHC) images. On the left side: a primary tumour which was HER2-low (1+) at diagnosis while its subcutaneous metastasis was HER2-negative (**a**, **c**: haematoxylin & eosin (H&E) staining; **b**, **d**: HER2 IHC). The opposite is shown on the right side: a HER2-negative primary breast tumour which relapsed as a HER2-low (1+) ovarian metastasis (**e**, **g**: H&E staining; **f**, **h**: HER2 IHC).

The HER2 status significantly differed between the primary and recurrent tumour in 191 cases (37.3%; *K* = 0.468, 95%CI 0.400–0.536), a majority of which (*n* = 157; 82.2%) were either HER2-negative, or HER2-low at diagnosis. Indeed, we observed an increased proportion of tumours with the HER2-low status among recurrences: 50.0% versus 44.9% at diagnosis, while only 33.8% of tumours were HER2-negative (compared to 39.3% which were HER2-negative at diagnosis). The HER2-low status was also more prevalent among HR-positive (luminal-like) than among HR-negative recurrent BCs (54.0% versus 29.1%; *P* < 0.001). However, the proportion of HER2-positive tumours remained stable (15.8% versus 16.2%). The changes in the HER2 status between primary and relapsed tumours in the study population stratified by the HR expression in the primary tumour are shown in Fig. 1, while Fig. 2 shows examples of typical immunohistochemical stainings.

### Changes in the HER2 status by type of recurrence

**Fig. 3:**
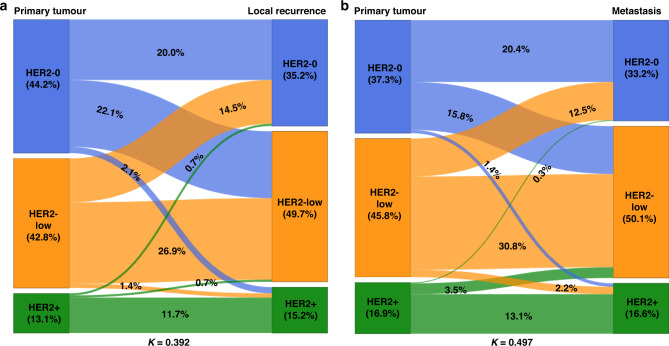
Evolution of HER2 status from primary breast tumour to matched recurrence according to the type of relapse. Sankey diagrams illustrating the HER2 status changes for distant metastases (*n* = 367) (**a**) and local recurrence (*n* = 145) (**b**), with specific percentages. HER2-0 HER2-negative.

Over two-thirds of the patients included in our study (367; 71.7%) had distant metastases, while 145 patients (28.3%) presented local recurrences. These two groups had similar rates of HER2-low primary tumours at diagnosis: 42.8% among patients who later developed locoregional recurrences and 45.8% among those who developed distant metastases (*P* = 0.603). We found that the proportion of recurrent tumours with changed HER2 status was significant in each of these two groups (*n* = 131 (35.7%); *K* = 0.497, 95% CI 0.419–0.575 for distant metastases and *n* = 60 (41.4%); *K* = 0.392, 95% CI 0.252–0.532 for local recurrences) but without a significant difference between them (*P* = 0.303). As in the whole study population, in each recurrence subgroup, changes were mostly observed for patients whose tumours were HER2-negative or HER2-low at diagnosis: 89.3% of metastatic tumours (117 cases) and 98.3% of local recurrences (59 cases) with a changed HER2 status developed from primary tumours which were HER2-negative or -low at diagnosis. Higher proportions of HER2-low recurrent tumours than primary tumours were observed in both groups: 50.1% for distant metastases versus 45.8% in the corresponding primary tumours, and 49.7% versus 42.8% for local recurrences. The changes in the HER2 status between primary and relapsed tumours according to the type of recurrence (distant or local) are shown in Fig. 3, and the changes stratified by HR expression in Supplementary Fig. 1. Detailed demographic, clinical, and histological data of patients and tumours according to the type of recurrence are shown in Supplementary Table 1. However, the patterns of the HER2 status changes did not significantly differed between metastatic sites.

### Factors associated with changes in the HER2 status

Since we found that changes in the HER2 status in recurrent tumours compared to primary tumours were particularly frequent in patients with HER2-negative and HER-low primary tumours, we compared main clinical and pathological characteristics of primary HER2-negative BCs stratified by the HER2 status change pattern between the primary and recurrent tumour (stable or not). The clinical, morphological and immunohistochemical characteristics of initially HER2-negative tumours, stratified by the HER2 status evolution, are summarised in Table 3. When analysing the HER2 status changes in the whole study population, we identified two main factors which were significantly associated with these changes. Indeed, HER2-negative tumours which gave rise to HER2-low recurrences more frequently expressed oestrogen receptors (ER) (88.9% versus 73.8%, *P* = 0.008) and recurred later (90.3 ± 54.5 versus 68.2 ± 51.9 months; *P* = 0.002) than HER2-negative primary tumours which gave rise to recurrences with unchanged HER2-negative status. These results were confirmed by multivariate analysis which identified ER expression (OR = 2.66; 95 CI: 1.12–6.29; *P* = 0.027) and time to recurrence (OR = 2.14; 95 CI: 1.15–4.01; *P* = 0.017) as two parameters independently associated with the risk of an increased HER2 expression in the recurrent tumour. Of note, no significant differences in any of the analysed parameters were found for HER2-low tumours which gave rise to HER2-negative recurrences.

**Table 3:** Clinical, pathological, and immunohistochemical characteristics of breast cancer patients and tumours for the subgroup with HER2-negative tumours at diagnosis, by HER2 status at recurrence (local recurrences and distant metastases together).

Parameter	Primary tumours which relapsed as HER2-low (*n* = 90)	Primary tumours which relapsed as HER2-negative (unchanged HER2 status) (*n* = 104)
Age at diagnosis (years)		
Mean ± SD	54.1 ± 11.4	57.3 ± 14.0
Median [min–max]	55.0 [26.0–81.0]	57.5 [26.0–89.0]
* P* value	*0.075*	
Unifocal tumour		
Yes	75 (83.3%)	85 (81.7%)
No	15 (16.7%)	19 (18.3%)
* P* value	*0.770*	
Histology		
IDC	65 (72.2%)	79 (76.0%)
ILC	22 (24.4%)	21 (20.2%)
Other	3 (3.3%)	4 (3.8%)
* P* value	*0.782*	
Tumour size (cm)		
Mean ± SD	2.2 ± 1.3	2.7 ± 2.2
Median [min–max]	1.9 [0.4–6.5]	2.0 [0.1–10.5]
Missing data	3	1
* P* value	*0.113*	
E&E grade		
I	20 (22.5%)	18 (17.8%)
II	48 (53.9%)	46 (45.5%)
III	21 (23.6%)	37 (36.6%)
Missing data	1	3
* P* value	*0.148*	
Glandular differentiation		
I	2 (2.2%)	3 (2.9%)
II	27 (30.3%)	24 (23.5%)
III	60 (67.4%)	75 (73.5%)
Missing data	1	2
* P* value	*0.577*	
Nuclear grade		
I	6 (6.7%)	7 (6.9%)
II	61 (68.5%)	60 (58.8%)
III	22 (24.7%)	35 (34.3%)
Missing data	1	2
* P* value	*0.337*	
Mitosis score		
I	48 (53.9%)	41 (40.2%)
II–III	41 (46.1%)	61 (59.8%)
Missing data	1	2
* P* value	*0.080*	
Lymph node status		
N0–N0i+	51 (56.7%)	52 (50.0%)
N1–N1mic	26 (28.9%)	34 (32.7%)
N2–N3	13 (14.4%)	18 (17.3%)
* P* value	*0.645*	
Disease stage		
I	39 (39.2%)	37 (35.6%)
II	31 (32.0%)	31 (29.8%)
III	19 (23.7%)	27 (26.0%)
IV	1 (5.2%)	9 (8.7%)
* P* value	*0.076*	
Oestrogen receptor expression		
Negative	10 (11.1%)	27 (26.2%)
Positive	80 (88.9%)	76 (73.8%)
Missing data	0	1
* P* value	***0.008***	
Progesterone receptor expression		
Negative	25 (30.9%)	35 (35.7%)
Positive	56 (69.1%)	63 (64.3%)
Missing data	9	6
* P* value	*0.494*	
Ki67 expression		
Mean ± SD	28.7 ± 19.6	32.8 ± 22.9
Median [min–max]	25.0 [1.0–80.0]	30.0 [1.0–90.0]
Missing data	66	78
* P* value	*0.501*	
Time to recurrence (months)		
Mean ± SD	90.3 ± 54.5	68.2 ± 51.9
Median [min–max]	80.0 [2.0–240.0]	53.5 [1.0–212.0]
* P* value	***0.002***	

*E&E* Elston and Ellis, *HER2* human epidermal growth factor receptor 2, *IDC* invasive ductal carcinoma, *ILC* invasive lobular carcinoma, *SD* standard deviation.

Values in bold italic show statistically significant *P*-values.

Then, in an attempt to identify factors which may predict changes in the HER2 status between primary and recurrent tumours, we analysed clinical, pathological and immunohistochemical characteristics for patients with HER2-negative primary tumours who developed local recurrences compared to those with distant metastases. We found that basic pathological and immunohistochemical characteristics of primary tumours which gave rise to recurrences with a changed HER2 status were different between these two groups. Indeed, for patients who presented with distant metastases, HER2-negative tumours which relapsed as HER2-low more frequently expressed ER (94.8% versus 73.0% among tumours which relapsed without a change in the HER2 status; *P* = 0.001), had significantly lower proliferation indexes (3.1 ± 3.6/mm^2^ versus 7.3 ± 9.6; *P* = 0.008) with lower mitosis scores (56.1% versus 35.6% with mitosis score I; *P* = 0.020) and lower Ki67 expression (28.7 ± 19.6% versus 32.8 ± 22.9%; *P* = 0.047). They also seemed to metastasise later (time to recurrence of 84.2 ± 49.8 versus 68.7 ± 51.1 months), but the trend was not statistically significant (*P* = 0.051). For HER2-low tumours which gave rise to HER2-negative recurrences, only one parameter was found to be different compared to tumours with a stable HER2 status (negative in the primary and in the recurrent tumour): a significantly higher Ki67 expression in the first tumour category (39.8 ± 20.2% versus 28.5 ± 17.1% for stable HER2-negative tumours; *P* = 0.037). Concerning local recurrences, HER2-negative tumours which gave rise to HER2-low recurrences relapsed significantly later than tumours with stable HER2 expression (101.3 ± 61.4 versus 66.9 ± 54.8 months; *P* = 0.013) and tended to appear in younger patients (52.4 ± 11.8 versus 60.8 ± 17.1 years), even though these differences were not statistically significant (*P* = 0.057). No significant differences were found between HER2-low tumours which relapsed as HER2-negative tumours and those which gave rise to recurrences with the same HER2 status (HER2-low). All these data are summarised in Table 4.

**Table 4:** Clinical, pathological and immunohistochemical characteristics of breast cancer patients and tumours for the subgroup with HER2-negative tumours at diagnosis, by changes in the HER2 status between the primary and recurrent tumour and by the type of relapse.

Parameter	Primary tumours which relapsed as local recurrences		Primary tumours which relapsed as distant metastases	
	HER2-low relapse (*n* = 32)	HER2-negative relapse (unchanged HER2 status) (*n* = 29)	HER2-low relapse (*n* = 58)	HER2-negative relapse (unchanged HER2 status) (*n* = 75)
Age at diagnosis (years)				
Mean ± SD	52.4 ± 11.8	60.8 ± 17.1	55.0 ± 11.1	56.0 ± 12.4
Median [min–max]	51.5 [26.0–79.0]	66.0 [34.0–89.0]	56.0 [29.0–81.0]	56.0 [26.0–87.0]
* P* values	*0.057*		*0.628*	
Unifocal tumour				
Yes	28 (87.5%)	27 (93.1%)	47 (81.0%)	58 (77.3%)
No	4 (12.5%)	2 (6.9%)	11 (19.0%)	17 (22.7%)
* P* values	*0.674*		*0.604*	
Histology				
IDC	26 (81.2%)	23 (79.4%)	39 (67.2%)	56 (74.7%)
ILC	6 (18.8%)	5 (17.2%)	16 (27.6%)	16 (21.3%)
Other	0 (0.0%)	1 (3.4%)	3 (5.2%)	3 (4.0%)
* P* values	*0.866*		*0.651*	
Tumour size (cm)				
Mean ± SD	1.6 ± 1.0	1.8 ± 1.1	2.5 ± 1.4	3.1 ± 2.4
Median [min–max]	1.3 [0.4–4.5]	1.5 [0.1–4.3]	2.0 [0.7–6.5]	2.3 [0.2–10.5]
Missing data	0	0	3	1
* P* values	*0.358*		*0.321*	
E&E grade				
I	8 (25.0%)	7 (24.1%)	12 (21.1%)	11 (15.3%)
II	14 (43.8%)	10 (34.5%)	34 (59.6%)	36 (50.0%)
III	10 (31.3%)	12 (41.4%)	11 (19.3%)	25 (34.7%)
Missing data	0	0	1	3
* P* values	*0.681*		*0.146*	
Glandular differentiation				
I	2 (6.2%)	2 (6.9%)	0 (0.0%)	1 (1.4%)
II	8 (25.0%)	7 (24.1%)	19 (33.3%)	17 (23.3%)
III	22 (68.8%)	20 (69.0%)	38 (66.7%)	55 (75.3%)
Missing data	0	0	1	2
* P* values	*1.000*		*0.279*	
Nuclear grade				
I	3 (9.4%)	4 (13.8%)	3 (5.3%)	3 (4.1%)
II	21 (65.6%)	13 (44.8%)	40 (70.2%)	47 (64.4%)
III	8 (25.0%)	12 (41.4%)	14 (24.6%)	23 (31.5%)
Missing data	0	0	1	2
* P* values	*0.260*		*0.662*	
Mitosis score				
I	16 (50.0%)	15 (51.7%)	32 (56.1%)	26 (35.6%)
II–III	16 (50.0%)	14 (48.3%)	25 (43.9%)	47 (64.4%)
Missing data	0	0	1	2
* P* values	*0.893*		***0.020***	
Mitotic index (/mm²)				
Mean ± SD	7.7 ± 8.0	7.3 ± 9.6	3.1 ± 3.6	7.3 ± 9.6
Median [min–max]	5.7 [0.4–28.2]	2.2 [0.4–27.0]	1.9 [0.4–16.0]	4.2 [0.4–50.0]
Missing data	12	10	23	24
* P* values	*0.573*		***0.008***	
Lymph node status				
N0–N0i+	23 (71.9%)	20 (69.0%)	28 (48.3%)	32 (50.0%)
N1–N1mic	8 (25.0%)	9 (31.0%)	18 (31.0%)	25 (32.7%)
N2–N3	1 (3.1%)	0 (0.0%)	12 (20.7%)	18 (17.3%)
* P* values	*0.570*		*0.802*	
Disease stage				
I	20 (62.5%)	17 (58.6%)	19 (32.8%)	20 (26.7%)
II	9 (28.1%)	6 (20.7%)	22 (37.9%)	25 (33.3%)
III	3 (9.4%)	6 (20.7%)	16 (27.6%)	21 (28.0%)
IV	0 (0.0%)	0 (0.0%)	1 (1.7%)	9 (12.0%)
* P* values	*0.509*		*0.157*	
Oestrogen receptor expression				
Negative	7 (21.9%)	7 (24.1%)	3 (5.2%)	20 (27.0%)
Positive	25 (78.1%)	22 (75.9%)	55 (94.8%)	54 (73.0%)
Missing data	0	0	0	1
* P* values	*0.834*		***0.001***	
Progesterone receptor expression				
Negative	8 (25.0%)	11 (39.3%)	17 (34.7%)	24 (34.3%)
Positive	24 (75.0%)	17 (60.7%)	32 (65.3%)	46 (65.7%)
Missing data	0	1	9	6
* P* values	*0.235*		*0.963*	
Ki67 expression				
Mean ± SD	42.5 ± 20.4	28.8 ± 26.6	28.7 ± 19.6	32.8 ± 22.9
Median [min–max]	40.0 [20.0–80.0]	20.0 [8.0–90.0]	25.0 [1.0–80.0]	30.0 [1.0–90.0]
Missing data	24	19	66	78
* P* values	*0.108*		***0.047***	
Metastatic site				
Liver	–	–	17 (29.3%)	19 (25.3%)
Bone	–	–	9 (15.5%)	18 (24.0%)
Skin and muscle	–	–	9 (15.5%)	15 (20.0%)
Lymph nodes	–	–	11 (19.0%)	7 (9.3%)
Lung and pleura	–	–	4 (6.9%)	6 (8.0%)
Gynaecological tract	–	–	3 (5.2%)	4 (5.3%)
Gastrointestinal tract	–	–	4 (6.9%)	1 (1.3%)
Brain	–	–	0 (0.0%)	1 (1.3%)
Other	–	–	1 (1.7%)	4 (5.3%)
* P* values	–		*0.402*	
Time to recurrence (months)				
Mean ± SD	101.3 ± 61.4	66.9 ± 54.8	84.2 ± 49.8	68.7 ± 51.1
Median [min–max]	88.0 [14.0–215.0]	48.0 [6.0–212.0]	75.0 [2.0–240.0]	57.0 [1.0–204.0]
* P* values	***0.013***		*0.051*	

*E&E* Elston and Ellis, *HER2* human epidermal growth factor receptor 2, *IDC* invasive ductal carcinoma, *ILC* invasive lobular carcinoma, *SD* standard deviation.

Values in bold italic show statistically significant *P*-values.

When focusing on patients with HR-positive primary tumours, i.e., the population for which the HER2 status changes were the most frequent, we found that the time to recurrence was also—as for the whole study population—the main factor associated with these changes, both for the whole group with HR-positive primary tumours and just those with HR-positive local recurrences (*P* = 0.014 and *P* = 0.006, respectively), and this was independent of the primary tumour HR status at diagnosis. Concerning HR-positive BCs which gave rise to distant metastases, we identified PR expression as the only factor which was significantly associated with changes in the HER2 status between the primary and recurrent tumour. Indeed, we found a lower proportion of tumours expressing PR among patients for whom the HER2 status differed between the primary and the recurrent tumour (HER2-negative in the primary tumour and HER2-low in the recurrence), and— inversely—a higher proportion of PR-expressing primary tumours was found among patients with stable HER-negative status (69.6% versus 90.2%; *P* = 0.010). However, despite an insignificant trend to lower mitotic indexes in patients with HER2-negative primary tumours which gave rise to HER2-low recurrences (*P* = 0.057), no significant differences in proliferation rates (mitosis scores and Ki67 expression) were found. All results of these comparisons are summarised in Supplementary Table 2.

## Discussion

HER2-low breast tumours, accounting for approximately half of all BCs, are defined as tumours with the HER2 IHC score of 1 + , or of 2+ with no HER2 amplification by ISH [19, 20]. These tumours represent a new subgroup of BC with a specific biology as well as different responsiveness to therapy and different prognosis [24]. The recognition of this novel entity is fortunately intertwined with the recent development of new anti-HER2 antibody-drug conjugate (ADC) therapies which may prove effective in patients with HER2-low tumours. Indeed, until recently, anti-HER2 treatments were exclusively reserved for patients with the *ERBB2* amplification (defined by HER2 IHC 3+ or 2+ with ISH positivity) and no clinical benefits were highlighted for patients with HER2-low tumours [17, 19, 25]. This paradigm has been totally reversed by the recent development of trastuzumab deruxtecan (DS-8201a or T-DXd) and trastuzumab duocarmazine (SYD985), two novel anti-HER2 agents acting through alternative pharmacological mechanisms including the delivery of targeted cytotoxic agents into cancer cells, with encouraging results obtained in patients with HER2-low tumours [16–19, 26].

The development of these new therapies has stirred up lots of interest in the characterisation of HER2-low tumours [19, 27]. However, little is known about the dynamics of the HER2 status changes between HER2-low primary tumours and their recurrences [21, 22]. In this study, we characterised the HER2 status differences between primary tumours and matched relapses (local recurrences and distant metastases). Moreover, we identified—for the first time ever reported in scientific literature—clinical and pathological factors that could predict these changes.

Our cohort comprised a total of 512 breast cancer patients for whom HER2 status data were available for both the primary tumour and a matched recurrence. Primary tumours which were HER2-low at diagnosis represented just under a half of the entire study sample (44.9%), followed by HER2-negative tumours (39.3%) and HER2-positive tumours (15.8%), which is concordant with the data reported in the literature [8, 19, 20]. The rates of HER2-low primary tumours were similar between patients who later developed distant metastases and those who developed locoregional recurrences.

Consistently with what was previously reported by others [20, 21, 24], we found a small but statistically significantly difference in the ER expression between HER2-low tumours (higher ER expression) compared to HER2-negative tumours at diagnosis. We also found that the HER2-low status was more prevalent among HR-positive (luminal-like) tumours than among HR-negative BCs (TNBCs), both at diagnosis and at recurrence. These findings are in line with the hypothesis of a crosstalk between the HER2 and HR pathways, and may partly explain the higher proportion of HER2-low status among HR-positive tumours [28–31]. However, further studies including larger numbers of patients are needed to confirm a possible association between the HER2-low and the HR status in primary breast tumours.

Our analyses showed a significant discordance in the HER2 expression between primary tumours and matched recurrences, with HER2 status changes found in more than one third of recurrent tumours (37.3%). These changes concerned mainly recurrences of HER2-negative tumours, with a higher proportion of HER2-low tumours in advanced stages of the disease (50% of recurrences compared to 44.9% among primary tumours). Indeed, while HER2-positive status was usually stable during disease progression, the recurrences of HER2-negative and HER2-low primary tumours often had a higher HER2 expression than the primary tumours they developed from. Shifts from HER2-negative towards HER2-low tumours were particularly common, especially in HR-positive (luminal-like) tumours, as it has been reported by previous studies [21, 22]. We found the same change patterns for local recurrences and distant metastases alike: the HER2 status of recurrent tumours significantly differed from that of matched primary tumours for both these groups but without a significant difference between them. In each relapse category, these changes mostly concerned recurrences of tumours which were HER2-negative or HER2-low at diagnosis, with an increase in the prevalence of the HER2-low status among recurrent tumours in both groups. However, no significant differences in the HER2 status change patterns were observed between different metastatic sites, contrary to reports by others [21].

The observed HER2 expression changes may be explained by a number of factors associated with the tumour and/or the diagnostic process. Firstly, they could be partly explained by intra-tumoral heterogeneity which was reported by several authors and which is probably associated with resistance to HER2-targeted therapy [32, 33]. Indeed, HER2 expression seems to be non-uniform in some breast tumours due to the co-existence of multiple cancer cell subpopulations in the same tumour area or in different areas of a single tumour, and so lead to discrepant HER2 status results, depending on the analysed region [33]. Then, BC treatments, whether surgical or systemic (including chemotherapy, hormone therapy or conventional anti-HER2 therapies), could modify (upregulate) the HER2 expression levels in tumour cells during the course of the disease [34–38]. Furthermore, many cases of the presumed HER2 expression change may be due to the evolution of HER2 immunohistochemistry protocols, including labelling heterogeneity depending on the reagents and antibodies used, and the introduction of quality controls over time [20, 26, 39, 40]. Indeed, before the development of ADC therapies, HER2 IHC 0 and 1+ scores were both considered to be HER2-negative. Since discriminating between the 0 and 1+ IHC score had no clinical implications, some laboratories may have used protocols which did not reliably differentiate between these two categories, and so it is possible that some of the 1+ specimens were simply misclassified. For example, the fact that no on-slide quality controls were used in the early 2000’s may have led to false negative results. These controls, progressively emerging over time with the improvement of staining protocols, are considered to be essential today. Indeed, both negative (0), positive (3 + ), or even—ideally—multiple (0; 1+; 2+; 3+) internal or external controls are now required to be used for all HER2 immunohistochemistry stainings as essential for result interpretation. Finally, some of the discordances in the HER2 status between the primary and recurrent tumours we observed in our study may be due to inter-observer variability, i.e., variations in the interpretation of HER2 testing results, especially when it comes to differentiating between HER2 IHC 0 and 1+ scores, which can be very challenging [20, 26, 39]. In a recent study, Schettini and colleagues evaluated the reproducibility of HER2 IHC scoring by five pathologists specialised in breast cancer, coming from different institutions, on a hundred cases, and showed that the reproducibility was suboptimal, with 35% of discordant cases, especially in the HER2-low subgroup [20]. However, in the era of anti-HER2 ADC therapies which are indicated for patients with HER2-low BCs, it is essential to develop more sensitive assays for HER2 evaluation, to harmonise scoring practices by training pathologists, and to strongly incite pathology laboratories to apply stringent quality control systems in order to better discriminate HER IHC 0 and 1+ specimens and so correctly identify HER2-low tumours so as to give a chance of treatment to all eligible patients.

Our finding that the HER2 status changes with disease progression, with a higher HER2 expression at relapse, is a very important result. Indeed, it may lead to increasing the proportion of patients who would be offered new anti-HER2 ADCs and who could benefit from these treatments, especially at advanced stages of the disease after the failure of multiple lines of treatment [18, 26, 41]. These statements are all the more true for patients with HR-positive BCs among whom the enrichment of HER2-low tumours in advanced disease is the most prevalent. It also constitutes a real opportunity for patients with triple-negative breast cancer (TNBC). These tumours are all HR-negative and HER2-negative by definition. However, this definition was coined before the category of HER2-low tumours was distinguished and so some of these seemingly HER2-negative tumours may actually be HER2-low. Even though the prevalence of the HER2-low status among TNBCs is lower than among other BC types, it is still not negligible. In our study, over a third of primary TNBCs were HER2-low at diagnosis (38.8%). Therefore, a possibility of identifying a subgroup of TNBC patients who may benefit from new anti-HER2 therapies is particularly promising, especially given that these tumours are usually associated with poor prognosis due to the scarcity of effective therapeutic options [42–45]. All these data strongly support the imperative need to retest breast tumours at relapse, regardless of the hormonal status of the primary tumour, in order to identify patients eligible for these new therapies.

Taking all this into account, and due to a large number of patients who could potentially benefit from new anti-HER2 treatments, particularly at relapse, we tried to identify factors possibly associated with the HER2 status changes during the course of the disease. As these changes mostly concerned recurrences of HER2-negative and HER2-low tumours, we compared main clinical and pathological characteristics of primary HER2-negative BCs, stratified by the HER2 status evolution category (stable or changed status)—it is the first time such a study has ever been reported in the literature. We highlighted two main factors significantly associated with HER2-negative tumours relapsing as HER2-low tumours in the whole study population: ER expression by the primary tumour and time to recurrence. We found that primary HER2-negative tumours which gave rise to HER2-low recurrences, more frequently expressed ER and recurred later than primary tumours which relapsed as HER2-negative. Concerning the link between HER2 status change and the time to (local) recurrence, one may also hypothesise that we observed newly arising primary tumours with a different HER2 status rather than real recurrences in which the HER2 status was different than in matched primary tumours. Time to recurrence seemed to be longer in patients who developed HER2-low recurrences also in case of distant metastases; however, this association was not statistically significant. Still, this similarity between the two groups argues against the hypothesis of a newly arising tumour rather than a relapse in case of local recurrences appearing long after the diagnosis of the primary tumour.

Interestingly, when analysing local recurrences and distant metastases separately, we identified different factors associated with HER2 status changes for each of the two categories. While ER expression in the primary tumour was an important factor predicting changed HER2 status in distant metastases, it was not so for local recurrences—only time to recurrence appeared to be associated with the HER2 status changes for the latter. Conversely, only high Ki67 expression seemed to be associated with decreased HER2 expression in distant metastasis, i.e., in the case of HER2-low primary tumours giving rise to HER2-negative distant metastases.

In accordance with the data reported in the literature [20, 21, 24], we found a strong association between the HER2-low status in recurrent tumours and HR expression in matched primary tumours, with a high proportion of HR-positive HER2-negative tumours that relapsed as HER2-low tumours. So, taking that into account, we then focused exclusively on patients with HR-positive primary tumours. We found that time to recurrence was the main predictive factor for HER2 status changes between primary and recurrent tumours among patients with HR-positive primary tumours which recurred locally, as was the case for all HR-positive tumours taken together. However, for HR-positive primary BCs which gave rise to distant metastases, only PR expression was significantly associated with HER2 status changes: a lower PR expression (ER+/PR- tumours) seemed to favour a change from HER2-negativity in the primary tumour to HER2-low status in the recurrence. This finding may have important clinical implications for patients with ER+/PR− HER2-negative tumours since their response to hormone therapies and their prognosis are known to be worse than those of patients with ER+/PR+ tumours [46–48]. In contrast to what we found for metastatic tumours, no significant differences in proliferation rates between tumours which relapsed with a changed HER2 status and those with unchanged HER2 expression were observed among patients with HR-positive primary tumours. This could be partly explained by a possible association between the HR status and the proliferation rate, as most HR-negative HER2-negative BCs (TNBCs) have high-proliferation indexes [45].

Our study has several limitations, mostly due to its single-centre retrospective design and especially because we restricted the study to BC patients with relapsed disease, which is not representative of the general population. Still, to the best of our knowledge, this is one of the largest studies investigating changes in the HER2 status between primary and recurrent breast tumours ever reported in the literature. In addition, our study is the first one to identify clinical and pathological factors predictive of changed HER2 status in recurrent tumours, making it possible to more accurately identify patients who may benefit from anti-HER2 ADC therapies.

In summary, in this study, we analysed the HER2 status changes between primary tumours and matched relapses, in 512 primary BC patients. We also characterised these tumours in clinical and pathological terms. We showed that HER2-low tumours are a specific entity, accounting for approximately half of all primary BCs, with particular biological features which seem to be closely related to HR expression (the proportion of HER2-low tumours is much higher among HR + than among HR− tumours). Moreover, we demonstrated a high prevalence of HER2 expression changes during the course of the disease, mainly enrichment of HER2-low tumours in the advanced disease stage. We also identified, for the first time ever reported in the literature, some clinical and pathological factors predictive of these changes. The fact that increased HER2 expression in recurrent tumours compared to primary tumours is relatively common strongly supports the need to retest for HER2 expression at relapse (whether local recurrence or distant metastases), particularly for patients with primary ER+/PR-tumours as well as those with, low proliferation indexes and late recurrences, in order to select the best candidates for new anti-HER2 ADC therapies.

## Supplementary information

Supplementary information

## Data Availability

All data analysed in this study are included in this article or in the associated additional files.
