# Supplementary material for: Anticipating changes in the HER2 status of breast tumours with disease progression—towards better treatment decisions in the new era of HER2-low breast cancers
Source: Br J Cancer. 2023 Apr 29;129(1):122–34. doi: 10.1038/s41416-023-02287-x (PMC10307899; doi:10.1038/s41416-023-02287-x)
Supplement: Supplementary file 1 — Supplementary information [file 41416_2023_2287_MOESM1_ESM.pdf]

Supplementary Table 1. Detailed demographic, clinical, and histological data of the study patients and tumours at diagnosis according to the type of recurrence

	**All (n = 512)**	**Primary tumours which gave rise to local recurrences (n = 145)**	**Primary tumours which gave rise to distant metastases (n = 367)**
Age at diagnosis (years)			
Mean ± SD	55.8 ± 12.9	56.9 ± 14.1	55.4 ± 12.4
Median [min-max]	56.0 [25.0 - 89.0]	56.0 [26.0 - 89.0]	55.0 [25.0 - 87.0]
P-value		*0.335*	
Unifocal tumour			
Yes	397 (77.5%)	123 (84.8%)	274 (79.3%)
No	115 (22.5%)	22 (15.2%)	93 (25.3%)
P-value		***0.013***	
Histology			
IDC	412 (80.5%)	121 (83.4%)	291 (80.0%)
ILC	91 (17.8%)	22 (15.2%)	69 (18.8%)
Other	9 (1.8%)	2 (1.4%)	7 (1.9%)
P-value		*0.560*	
Tumour Size (cm)			
Mean ± SD	2.7 ± 1.9	1.9 ± 1.4	3.0 ± 1.9
Median [min-max]	2.2 [0.1 – 10.5]	1.5 [0.1 – 10]	2.5 [0.2 – 10.5]
Missing data	6	0	6
P-value		**< *0.001***	
E&E grade			
I	83 (16.4%)	38 (26.4%)	45 (12.5%)
II	264 (52.3%)	62 (43.1%)	202 (56.0%)
III	158 (31.3%)	44 (30.6%)	114 (31.6%)
Missing data	7	1	6
P-value		**< *0.001***	
Glandular differentiation			
I	7 (1.4%)	6 (4.2%)	1 (0.3%)
II	146 (28.9%)	49 (34.0%)	97 (26.9%)
III	352 (69.7%)	89 (61.8%)	263 (72.9%)
Missing data	7	1	6
P-value		**< *0.001***	
Nuclear grade			
I	18 (3.6%)	10 (6.9%)	8 (2.2%)
II	330 (65.3%)	93 (64.6%)	237 (65.7%)
III	157 (31.1%)	41 (28.5%)	116 (32.1%)
Missing data	7	1	6
P-value		***0.032***	
Mitosis score			
I	237 (46.9%)	78 (54.2%)	159 (44.0%)
II	124 (24.6%)	25 (17.4%)	99 (27.4%)
III	144 (28.5%)	41 (28.5%)	103 (28.5%)
Missing data	7	1	6
P-value		***0.039***	
Lymph node status			
N0-N0i+	235 (45.9%)	103 (71.1%)	132 (36.0%)
N1-N1mic	188 (36.7%)	37 (25.5%)	151 (41.1%)
N2-N3	89 (17.4%)	5 (3.4%)	84 (22.9%)
P-value		**< *0.001***	
Disease stage			
I	165 (32.2%)	86 (59.3%)	79 (21.5%)
II	179 (35.0%)	30 (20.7%)	149 (40.6%)
II	128 (25.0%)	27 (18.6%)	101 (27.5%)
IV	40 (7.8%)	2 (1.4%)	38 (10.4%)
P-value		**< *0.001***	
Oestrogen receptor expression			
Positive	413 (81.6%)	115 (79.9%)	298 (82.3%)
Negative	93 (18.4%)	29 (20.1%)	64 (17.7%)
Missing data	6	1	5
P-value		*0.519*	
Progesterone receptor expression			
Positive	322 (66.4%)	98 (69.0%)	224 (76.7%)
Negative	163 (33.6%)	44 (31.0%)	119 (34.7%)
Missing data	27	3	24
P-value		*0.432*	
HER2 status (IHC+/-FISH)			
0	201 (39.3%)	64 (44.1%)	137 (37.3%)

1+	155 (30.3%)	44 (30.3%)	111 (30.2%)
2+ non-amplified	75 (14.6%)	18 (12.4%)	57 (15.5%)
2+ amplified	14 (2.7%)	1 (0.7%)	13 (3.5%)
3+	67 (13.1%)	18 (12.4%)	49 (13.4%)
P-value		*0.279*	
Time to recurrence (months)			
Mean ± SD	72.3 ± 50.8	88.2 ± 56.1	66.0 ± 47.2
Median [min-max]	60 [1.0 - 240.0]	77.0 [6.0 - 235.0]	60 [1.0 - 240.0]
P-value		***< 0.001***	
HER2 status change between primary and recurrent tumour			
Stable HER2-negative	104 (20.3%)	29 (20.0%)	75 (20.4%)
Stable HER2-low	152 (29.7%)	39 (26.9%)	113 (30.8%)
Stable HER-positive	65 (12.7%)	17 (11.7%)	48 (13.1%)
HER2-negative to HER2-low	90 (17.6%)	32 (22.1%)	58 (15.8%)
HER2-negative to HER2-positive	8 (1.6%)	3 (2.1%)	5 (1.4%)
HER2-low to HER2-negative	67 (13.1%)	21 (14.5%)	46 (12.5%)
HER2-low to HER2-positive	10 (2.0%)	2 (1.4%)	8 (2.2%)
HER2-positive to HER2-negative	2 (0.4%)	1 (0.7%)	1 (0.3%)
HER2-positive to HER2-low	14 (2.7%)	1 (0.7%)	13 (3.5%)
P-value		*0.303*	

E&E: Elston and Ellis; FISH: fluorescent in situ hybridisation; HER2: human epidermal growth factor receptor 2; IDC: invasive ductal carcinoma; IHC: immunohistochemistry; ILC: invasive lobular carcinoma; SD: standard deviation
In bold: *P*-values indicative of a statistical significance

Supplementary Table 2. Clinical, pathological, and immunohistochemical characteristics of breast cancer patients and tumours for the subgroup with HR-positive/HER2-negative tumours at diagnosis, by changes in the HER2 status and the type of relapse

	All		Local recurrences		Distant metastases	
	**Tumours which relapsed as HER2-low (n = 80)**	**Tumours which relapsed as HER2-negative (unchanged HER2 status) (n = 77)**	**Tumours which relapsed as HER2-low (n = 25)**	**Tumours which relapsed as HER2-negative (unchanged HER2 status) (n = 22)**	**Tumours which relapsed as HER2-low (n = 55)**	**Tumours which relapsed as HER2-negative (unchanged HER2 status) (n = 55)**
**Parameter**						
Age at diagnosis (years)						
Mean ± SD	55.2 ± 10.9	57.8 ± 14.0	55.8 ± 10.1	63.2 ± 17.5	55.0 ± 11.3	55.6 ± 11.8
Median [min-max]	56.0 [29.0 - 81.0]	57.0 [34.0 - 89.0]	56.0 [38.0 - 79.0]	70.5 [34.0 - 89.0]	56.0 [29.0 - 81.0]	55.0 [36.0 - 87.0]
P-values	0.207		*0.075*		*0.780*	
Unifocal tumour						
Yes	66 (82.5%)	62 (80.5%)	22 (88.0%)	20 (90.9%)	44 (80.0%)	42 (76.4%)
No	14 (17.5%)	15 (19.5%)	3 (12.0%)	2 (9.1%)	11 (20.0%)	13 (23.6%)
P-values	*0.749*		*1.000*		*0.644*	
Histology						
IDC	56 (70.0%)	54 (70.1%)	19 (76.0%)	16 (72.7%)	37 (67.3%)	38 (69.1%)
ILC	22 (27.5%)	20 (26.0%)	6 (24.0%)	5 (22.7%)	16 (29.1%)	15 (27.3%)
Other	2 (2.5%)	3 (3.9)	0 (0.0%)	1 (4.5%)	2 (3.6%)	2 (3.6%)
P-values	*0.909*		*0.859*		*1.000*	
Tumour Size (cm)						
Mean ± SD	2.1 ± 1.3	2.7 ± 2.3	1.2 ± 0.6	1.6 ± 1.0	2.5 ± 1.4	3.2 ± 2.5
Median [min-max]	1.8 [0.4 – 6.5]	2.0 [0.1 – 10.5]	1.1 [0.4 – 2.8]	1.4 [0.1 - 4.3]	2.0 [0.7 - 6.5]	2.3 [0.2 - 10.5]
Missing data	3	1	0	0	3	1
P-value	*0.162*		*0.253*		*0.351*	
E&E grade						
I	20 (25.0%)	17 (22.6%)	8 (32.0%)	7 (31.8%)	12 (21.8%)	10 (18.9%)
II	45 (56.3%)	38 (50.7%)	13 (52.0%)	10 (45.5%)	32 (58.2%)	28 (52.8%)
III	15 (18.8%)	20 (26.7%)	4 (16.0%)	5 (22.7%)	11 (20.0%)	15 (28.3%)
Missing data	0	2	0	0	0	2
P-values	*0.500*		*0.926*		*0.598*	
Glandular differentiation						
I	2 (2.5%)	2 (2.6%)	2 (8.2%)	2 (9.1%)	0 (0.0%)	0 (0.0%)
II	26 (32.5%)	21 (27.6%)	8 (32.0%)	6 (27.3%)	18 (32.7%)	15 (27.8%)
III	52 (65.0%)	53 (69.7%)	15 (60.0%)	14 (63.6%)	37 (67.3%)	39 (72.2%)
Missing data	0	1	0	0	0	1
P-values	*0.848*		*1.000*		*0.574*	
Nuclear grade						
I	6 (7.5%)	7 (9.2%)	3 (12.0%)	4 (18.2%)	3 (5.5%)	3 (5.6%)
II	58 (72.5%)	52 (68.4%)	19 (76.0%)	13 (59.1%)	39 (70.9%)	39 (72.2%)
III	16 (20.0%)	17 (22.4%)	3 (12.0%)	5 (22.7%)	13 (23.6%)	12 (22.2%)
Missing data	0	1	0	0	0	1
P-values	*0.847*		*1.000*		*1.000*	
Mitosis score						
I	48 (60.0%)	39 (51.3%)	16 (64.0%)	15 (68.2%)	32 (58.2%)	24 (44.4%)
II-III	32 (40.0%)	37 (48.7%)	9 (36.0%)	7 (31.8%)	23 (41.8%)	30 (55.6%)

Missing data	0		1	0		0	0		1
P-values		*0.275*			*0.763*			*0.151*	
Mitotic index (/mm²)									
Mean ± SD	3.5 ± 4.3		5.3 ± 6.8	4.7 ± 5.4		3.7 ± 4.4	3.0 ± 3.7		6.0 ± 7.6
Median [min-max]	1.9 [0.4 – 16.0]		0.4 [0.4 – 33.0]	1.7 [0.4 - 15.5]		1.7 [0.4 - 16.5]	1.9 [0.4 - 16.0]		3.8 [0.4 - 33.0]
Missing data	33		24	11		6	22		18
P-values		*0.117*			*0.786*			*0.057*	
Lymph node status									
N0-N0i+	47 (58.8%)		39 (50.7%)	20 (80.0%)		16 (72.7%)	27 (49.1%)		23 (41.8%)
N1-N1mic	20 (25.0%)		23 (29.9%)	4 (16.0%)		6 (27.3%)	16 (29.1%)		17 (30.9%)
N2-N3	13 (16.2%)		15 (19.4%)	1 (4.0%)		0 (0.0%)	12 (21.8%)		15 (27.3%)
P-values		*0.595*			*0.851*			*0.710*	
Disease stage									
I	37 (46.3%)		31 (40.3%)	19 (76.0%)		14 (63.7%)	18 (32.7%)		17 (30.9%)
II	26 (32.5%)		19 (24.7%)	5 (20.0%)		5 (22.7%)	21(38.2%)		14 (25.5%)
III	16 (20.0%)		19 (24.7%)	1 (4.0%)		3 (13.6%)	15 (27.3%)		16 (29.1%)
IV	1 (1.3%)		8 (10.4%)	0 (0.0%)		0 (0.0%)	1 (1.8%)		8 (14.5%)
P-values		*0.062*			*0.406*			*0.075*	
Oestrogen receptor expression									
Negative	0 (0.0%)		1 (1.3%)	0 (0.0%)		0 (0.0%)	0 (0.0%)		1 (1.8%)
Positive	80 (100%)		76 (98.7%)	25 (100%)		22 (100%)	55 (100%)		54 (98.2%)
Missing data	*0*		1	0		0	0		1
P-values		*0.490*			*1.000*			*1.000*	
Progesterone receptor expression									
Negative	15 (21.1%)		9 (12.5%)	1 (4.0%)		4 (19.0%)	14 (30.4%)		5 (9.8%)
Positive	56 (78.9%)		63 (87.5%)	24 (96.0%)		17 (81.0%)	32 (69.6%)		46 (90.2%)
Missing data	9		5	0		1	9		4
P-values		*0.168*			*0.163*			***0.010***	
Ki67 expression									
Mean ± SD	22.9± 14.5		27.8 ± 19.4	30.0 ± 9.4		25.3 ± 25.7	20.5 ± 15.3		29.7 ± 14.0
Median [min-max]	23.5 [1.0 - 50.0]		25.0 [1.0 - 90.0]	25.0 [20.0 - 40.0]		20.0 [8.0 - 90.0]	15.0 [1.0 - 50.0]		30.0 [1.0 - 50.0]
Missing data	60		56	20		13	40		43
P-values		*0.479*			*0.177*			*0.123*	
Metastatic site									
Liver	-		-	-		-	16 (29.1%)		14 (25.5%)
Bone	-		-	-		-	9 (16.4%)		16 (29.1%)
Skin and muscle	-		-	-		-	9 (16.4%)		7 (12.7%)
Lymph nodes	-		-	-		-	10 (18.2%)		6 (10.9%)
Lung and pleura	-		-	-		-	4 (7.3%)		4 (7.3%)
Gynaecological tract	-		-	-		-	3 (5.5%)		4 (7.3%)
Gastrointestinal tract	-		-	-		-	3 (5.5%)		0 (0.0%)
Brain	-		-	-		-	0 (0.0%)		0 (0.0%)
Other	-		-	-		-	1 (1.8%)		4 (7.3%)
P-values		-			-			*0.344*	

Time to recurrence (months)						
Mean ± SD	93.7 ± 52.8	73.8 ± 52.2	108.4 ± 57.8	67.0 ± 52.5	87.0 ± 49.5	76.5 ± 52.3
Median [min-max]	84.0 [2.0 - 240.0]	60.0 [1.0 - 212.0]	110.0 [18.0 - 215.0]	49.0 [6.0 - 212.0]	84.0 [2.0 - 240.0]	60.0 [1.0 - 204.0]
P-values	***0.014***		***0.006***		*0.275*	

BC: breast cancer; E&E: Elston and Ellis; HER2: human epidermal growth factor receptor 2; HR: hormone receptor; IDC: invasive ductal carcinoma; ILC: invasive lobular carcinoma; SD: standard deviation

In bold: *P*-values indicative of a statistical significance

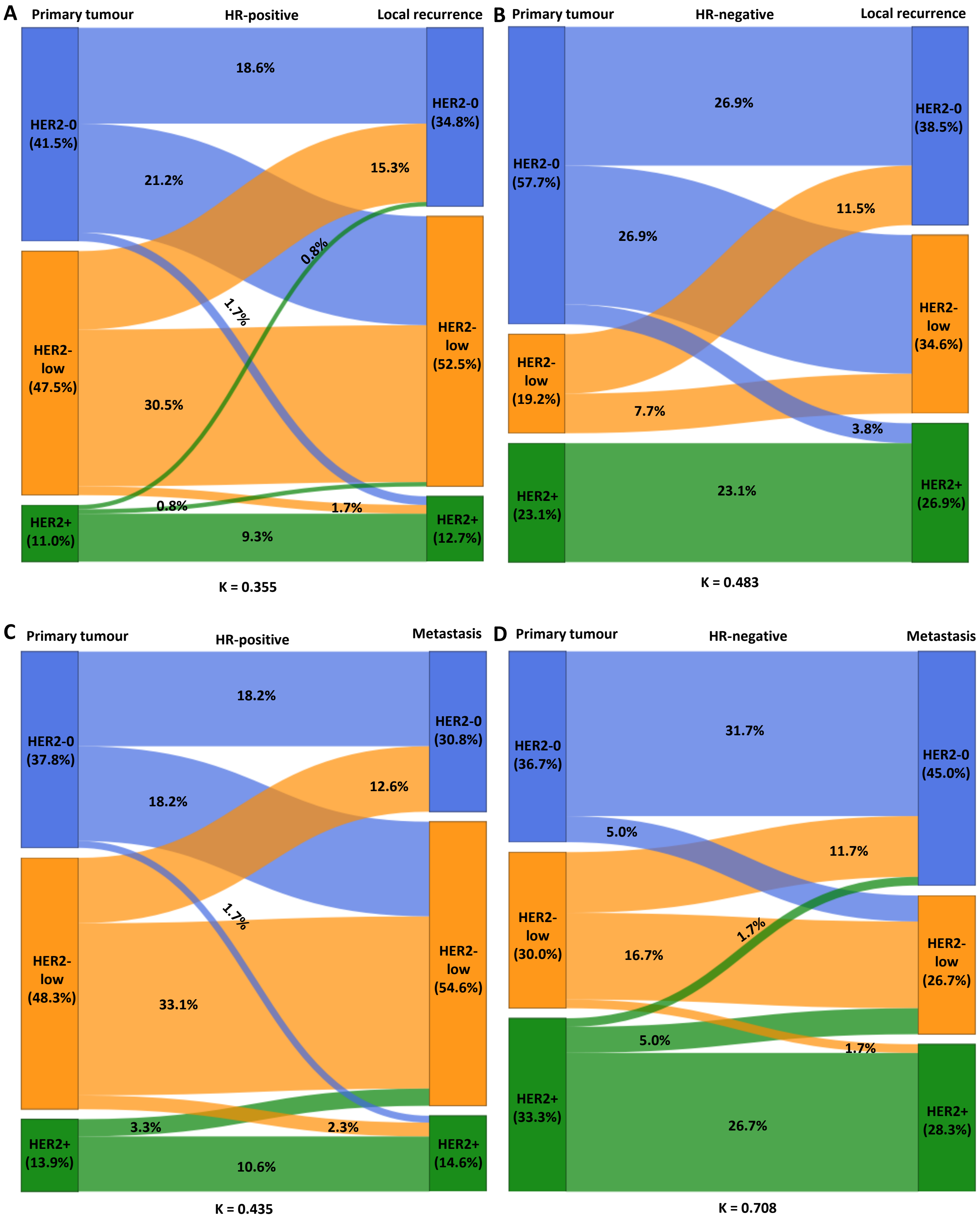

Supplementary Figure 1. **Changes in the HER2 status between primary breast tumours and matched relapses (local or distant recurrence), stratified by the hormone receptor (HR) expression.** Sankey diagrams illustrating the HER2 status changes for local recurrence **(A-B)** and distant metastases **(C-D)** according to the HR status, with specific percentages. HER2-0: HER2-negative, HER2+: HER2-positive
